# Molecular Characterization and Expression Analysis of the Na^+^/H^+^ Exchanger Gene Family in *Capsicum annuum* L.

**DOI:** 10.3389/fgene.2021.680457

**Published:** 2021-09-02

**Authors:** Xirong Luo, Shimei Yang, Yong Luo, Huarong Qiu, Tangyan Li, Jing Li, Xiaocui Chen, Xue Zheng, Yongdui Chen, Jie Zhang, Zhongkai Zhang, Cheng Qin

**Affiliations:** ^1^Department of Modern Agriculture, Zunyi Vocational and Technical College, Zunyi, China; ^2^Key Lab of Zunyi Crop Gene Resource and Germplasm Innovation, Zunyi Academy of Agricultural Sciences, Zunyi, China; ^3^Yunnan Provincial Key Lab of Agricultural Biotechnology, Key Lab of Southwestern Crop Gene Resources and Germplasm Innovation, Biotechnology and Germplasm Resources Institute, Yunnan Academy of Agricultural Sciences, Ministry of Agriculture, Kunming, China; ^4^School of Agriculture, Yunnan University, Kunming, China

**Keywords:** *Capsicum annuum*, CaNHX, phylogenetic tree, co-expression, abiotic stress

## Abstract

The Na^+^/H^+^ exchangers (*NHXs*) are a class of transporters involved in ion balance during plant growth and abiotic stress. We performed systematic bioinformatic identification and expression-characteristic analysis of *CaNHX* genes in pepper to provide a theoretical basis for pepper breeding and practical production. At the whole-genome level, the members of the *CaNHX* gene family of cultivated and wild pepper were systematically identified using bioinformatics methods. Sequence alignment and phylogenetic tree construction were performed using MEGA X software, and the gene functional domain, conserved motif, and gene structure were analyzed and visualized. At the same time, the co-expression network of *CaNHX* genes was analyzed, and salt-stress analysis and fluorescence quantitative verification of the Zunla-1 cultivar under stress conditions were performed. A total of 9 *CaNHX* genes were identified, which have typical functional domains of the Na^+^/H^+^ exchanger gene. The physical and chemical properties of the protein showed that the protein was hydrophilic, with a size of 503–1146 amino acids. Analysis of the gene structure showed that Chr08 was the most localized chromosome, with 8–24 exons. *Cis*-acting element analysis showed that it mainly contains *cis*-acting elements such as light response, salicylic acid response, defense, and stress response. Transcriptom and co-expression network analysis showed that under stress, the co-expressed genes of *CaNHX* genes in roots and leaves were more obvious than those in the control group, including ABA, IAA, and salt. The transcriptome and co-expression were verified by qRT-PCR. In this study, the *CaNHX* genes were identified at the genome level of pepper, which provides a theoretical foundation for improving the stress resistance, production, development, and utilization of pepper in genetic breeding.

## Introduction

Pepper (*Capsicum annuum* L.), also known variously as capsicum, chili pepper, chile, and chili, is an annual or perennial plant belonging to the Solanaceae family. It is one of the most important vegetable crops in the world (Qin et al., 2015). *Capsicum* species were first introduced into China during the Ming Dynasty and today, China has the largest planting area and fresh yield in the world (FAO).^1^ It is an important cash crop with many varieties, and is considered also of ornamental value, with considerable genetic diversity for research purposes and breeding (Zhang et al., 2016). Many varieties—including Zunla-1, Yunnan Xiaomi Spicy, and Hainan Bell Pepper—are widely planted in China, and their market share is increasing every year.

Peppers contain many substances of nutritional value including vitamin C and vitamin A. The fruits are not only used for food seasoning, but also in the production of food pigments, medicine, and industrial chemicals (Kantar et al., 2016). In medicine, it is widely used for multiple functions, including antibiosis and the prevention and treatment of disease (Saleh et al., 2018). Three pepper genomes (Zunla-1, Chiltepin, and CM334) have been completely sequenced, and with continuing re-sequencing, transcriptome sequencing, and metabolomics based on the whole genome, an increasing amount of genetic data of various pepper varieties has been mined (Kim et al., 2014; Qin et al., 2014). A key component of peppers is their capsaicin. Peppers produced in northwest China contain higher capsaicin and heme levels, due to the dry climate, low rainfall, high solar radiation, and wide temperature difference between day and night (Liu et al., 2012). This study examines how the nutritional content and capsaicin levels in peppers change when *Capsicum* is stressed by its growing environment. Under drought conditions, the capsaicin content in pepper can be reduced; under certain salt conditions, a significantly higher concentration of salt can promote the yield of capsaicin compared with control and low-salt pepper growth, and photosynthetic efficiency does not necessarily increase with salt (Sarah et al., 2012; Khan et al., 2014).

Plant growth and development depend strongly on environmental factors, such as cold and heat, drought, soil salinity and alkalinity, and other abiotic stresses. When plants are under stress, including some major cash crops, the external environment directly affects plant production. Salt stress is one of the most serious abiotic stresses affecting plant productivity and causes significant crop loss worldwide (Zhang et al., 2018). When plants are in a saline-alkaline soil environment, their ion balance and water balance change significantly. A change in membrane permeability destroys the normal operation of transporters, causing plants to absorb additional sodium ions from the environment, affecting the absorption of other ions and causing nutritional imbalance. Na^+^/H^+^ antiporters play a key role in plant development and tolerance to salt stress (Akram et al., 2020). In response to the external influence on plants, the ions and water in plants are balanced through their own ion channels. In general, the cytoplasmic pH value is above neutral (pH 7.2–7.6), which is controlled by an array of regulating molecules such as Na^+^/K^+^ transporters, cation/proton exchangers like Ca^2+^/H^+^, sodium-proton antiporters (NHX), proton/nutrient transporters, and H^+^-translocating enzymes (Benèina et al., 2009). Studies indicate that NHX antiporters are involved in regulating the ion balance in plants under salt stress. Their primary physiological functions are the regulation of cytoplasmic pH and expulsion of H^+^ generated during metabolism, in exchange for transporting Na^+^ or K^+^ ions into the cytoplasm and vacuoles of plants and animals (Pedersen et al., 2006). This indicates that studying the salt-tolerance mechanism of plants can improve the growth of plants under salt and alkali stress.

Human *HsNHE* was the first eukaryotic sodium-hydrogen exchanger gene to be identified and cloned, and functions in transport, Na^+^/H^+^ exchange, and pH regulation (Sardet et al., 1989). The first *NHX* gene identified in plants was the *AtNHX1* gene from *Arabidopsis thaliana*, and the expression of this gene can regulate NaCl in *A. thaliana* and is a salt-tolerance determiner (Gaxiola et al., 1999; Yokoi et al., 2002; Rodríguez-Rosales et al., 2009). Eight *NHX* genes were identified in *A. thaliana*, among which *AtNHX1* and *AtNHX2* were most common in the buds and roots of seedlings, while *AtNHX5* mRNA was expressed in lower abundance in both buds and roots. *AtNHX3* was detected in roots, while *AtNHX4* and *AtNHX6* mRNA were only detected by RT-PCR (Yokoi et al., 2002). To date, *NHX* genes of several plant species have been identified, such as *Vitis vinifera* (6 *VvNHX* genes) (Ayadi et al., 2020), *Medicago truncatula* (*MtNHX1*- *MtNHX8*) (Sandhu et al., 2018), *Populus trichocarpa* (*PtNHX1*- *PtNHX8*) (Tian et al., 2017), *Populus euphratica* (*PeNHX1*- *PeNHX6*) (Ye et al., 2009), *Gossypium hirsutum* (*GhNHX1*- *GhNHX23*) (Fu et al., 2020), *Morus alba* (*MaNHX1*- *MaNHX7*) (Cao et al., 2016), and *Beta vulgaris* (*BvNHX1*- *BvNHX5*) (Wu et al., 2019). This study performed different bioinformatic analyses of *CaNHX* genes in cultivated and wild peppers, and the *CaNHX* gene family of pepper was identified at the genome level, providing a theoretical basis for analyzing the function of the gene under salt stress.

## Materials and Methods

### Material and RNA Extraction

In this study, the whole genome data of pepper (*C. annuum* L. Zunla-1 is hereinafter referred to as pepper) and *C. annuum* var. glabriusculum Chiltepin were taken as the research object.^2^ Zunla-1 pepper material was planted in the greenhouse of the Department of modern agriculture, Zunyi vocational and technical college (Zunyi, Guizhou, 107°045 ’E, 27°710’ N). The pepper was treated with 100 Mmol NaCl for 3, 6, 12, 24, and 72 h. The root material of pepper was stored in liquid nitrogen, and 3 samples were taken at each time point as biological replicates. RNA was extracted from the collected samples using the TianGene RNA Extraction Kit (DP432, Beijing, China). We then added root material with a weight of 50–100 ng for aseptic freezing grinding; 450 μL for oscillation mixing. This was transferred to the CS filter column and centrifuged for 3 min (12,000 rpm). The supernatant was transferred from the collection tube with a pipette gun to the Rnase-free centrifuge tube. Then, the supernatant was added and 0.5 times of anhydrous ethanol was mixed into the centrifuge tube and then transferred to the adsorption column CR3 for centrifugation for 30 s (12,000 rpm). A drop of 80 μl DNase I was added to the center of the collecting tube and left at room temperature for 15 min. 250 μL of protein-removing solution RW1 was added to the adsorption column CR3, and left to stand at room temperature for 2 min, before being centrifuged for 30 s (12,000 rpm) (this procedure was repeated once). We then took an enzyme-free centrifuge tube and placed the adsorption column in a new centrifuge tube for several minutes (until the rinsing solution RW was dried). 50 μL Rnase-free ddH_2_O was then vertically added to the adsorption column, and the obtained RNA was stored at –80°C for later use.

### Identification of the *CaNHX* Gene in Pepper

*C. annuum* cv. CM334, *C. chinense* PI159236, *C. annuum* cv. ECW, *C. annuum* SF and *C. baccatum* PBC81 genomes come from PGP (Pepper Genome Platform).^3^ *Cuneo*, *Corno di Carmagnola*, *Quadrato di Carmagnola*, and *Tumaticot* genomes comes from RisEPP (Resequencing Piedmontese Pepper Ecotypes).^4^ According to the characteristics of the *NHX* gene family in Pfam^5^ data, there is an obvious conservative structure of the *NHX* gene family (PF00999) (El-Gebali et al., 2018). The genome-wide protein sequence of capsicum was searched by HMMER V3.3 software and verified with the Hmmer web server,^6^ and the sequences with the incomplete conservative structure were removed. meanwhile, the *AtNHX* gene of *A. thaliana* was used for blast comparison, and the *E*-value was maintained at 1e^–20^ for comparison. Selecting the intersection of HMMER identification and BLAST alignment, 9 candidate genes of *CaNHX* were finally identified for subsequent analysis (Potter et al., 2018). The physicochemical properties of the pepper *CaNHX* protein were analyzed using the online tool ExPASy^7^ (Artimo et al., 2012). Prediction of Plant-mPLoc by subcellular localization of the *CaNHX* gene in pepper was performed using online tools^8^ (Chou and Shen, 2010).

### Analysis of Phylogeny and Characteristics of the *CaNHX* Genes Family

MEGA X was used to perform multiple sequence alignment analysis on the obtained 9 pepper NHX protein sequences obtained, and the phylogenetic tree (neighbor-joining, bootstrap = 1,000) was constructed, and other parameters were left at default settings (Kumar et al., 2018). The online tool Itol^9^ was used for the presentation and form of the pepper *NHX* phylogenetic tree (Letunic and Bork, 2019). The batch CD-search^10^ tool in NCBI was used to visually analyze the *NHX* gene structure of the *NHX* gene. The online tool GSDS^11^ was used for the visualization of pepper *NHX* gene structure (Hu et al., 2014). The online sequence analysis tool MEME Suite^12^ was used for motif analysis, with the motif number set at 10 (Bailey et al., 2009). Collinearity analysis of pepper was performed by BLAST for whole-genome protein levels, and the MCScanX tool was used for collinearity analysis (Wang et al., 2012). TBtools were used for the visualization of gene structure, motifs, and collinearity results (Chen et al., 2020).

### Ka/Ks and Promoter Analysis of the *CaNHX* Genes Family

Using BLAST to build a pepper comparison database, and the KaKs Calculator tool to calculate the synonymous substitution rate and nonsensical substitution rate of pepper *CaNHX* genes, the K_*a*_/K_*s*_ ratio of genes was obtained, and evolutionary pressure was analyzed (Wang et al., 2010). The upstream 2,000 bp sequences of *NHX* genes were compared and extracted using the Bedtools genome analysis tool.^13^ The upstream 2,000 bp sequence was predicted and analyzed using the online tool PlantCARE.^14^ Visualization was performed with TBtools, the main action components were discussed (Lescot et al., 2002; Quinlan and Hall, 2010).

### Expression Model and Coexpression Analysis

Transcription factors (TFs) in the *C. annuum* genome were identified using the online iTAK Plant Transcription factor and Protein Kinase Identifier and Classifier (Zheng et al., 2016). The expression data^15^ obtained from pepper informatic hub were analyzed using temporal and spatial expression patterns and co-expression network associations. The root and leaf tissues of the CM334 pepper cultivar were used for transcriptome and metabolomic analysis, with a total of 574 transcriptome data points. The co-expression results were visualized using Cytoscape 3.7.2 (Liu et al., 2017; Otasek et al., 2019).

### cDNA Synthesis and Quantitative RT-PCR Analysis

The First Strand of RNA was synthesized using the Revertaid First Strand cDNA Synthesis Kit (K1622) from Thermo Field (RevertAid First Strand cDNA Synthesis Kit). The fluorescent quantitative primer Actin (GenBank: DQ832719) and Ubiquitin (GenBank: AY496112) were designed by Primer3plus software as the housekeeping gene (Supplementary Table 1). The fluorescence quantitative instrument for 15 samples was 96 Real-time qTOWER3.0 (Analytikjena, Germany), The fluorescence quantitative reaction system consisted of 10 μL SYBR Primix Ex Taq TM II (ZomBio, Beijing, China), and the upstream and downstream primers of each gene were 1 μL. And ddH2O to 20 μL. The PCR reaction procedure was 95°C for 30 s;95°C for 15 s;60°C for 30 s; and 72°C for 1 min, for 40 cycles. The quantitative RT-PCR results were analyzed using the 2^–△△^^Ct^ method (Livak and Schmittgen, 2001). GraphPad Prism v8 was used to visualize the fluorescence quantitative results.

## Results

### Identification and Physicochemical Properties of *CaNHX* Gene Family

According to the characteristics of the *NHX* gene family in the Pfam database, it contains Na_H_Exchanger (PF00999) functional domain. First, a total of 42 *NHX* genes were identified in pepper by the Hmmsearch identification method. Then, 8 *AtNHX* genes of *A. thaliana* were compared with the pepper genome by the BLASTP method. Combined with the two identification methods, the incomplete genes were removed by using the Hmmer online website. Nine *NHX* genes were obtained for subsequent analysis. These 9 *CaNHX* genes sequences were used for subsequent analysis and named *CaNHX1*-*CaNHX9* in turn (Supplementary Table 2 and Table 1). The physical and chemical properties of the protein showed that the size was 360–1181 aa, the molecular weight was 398.06–129.91 kDa, the isoelectric value was 5.44–8.79, GRAVY was less than 1, and it was a hydrophilic protein. After subcellular localization of pepper *CaNHX* gene, it was found that the subcellular localization of CaNHX2 and CaNHX4 was in the Cell membrane, and the other 7 subcellular localization were all in Vacuole.

**TABLE 1:** Family information and subcellular localization of *CaNHX* gene in pepper.

Gene_id	Gene_name	Chr	aa	Kd	pI	GRAVY	Sub. localization
Capana01g003109	*CaNHX1*	Chr01	511	56256.55	6.56	0.474	Vacuole
Capana05g000031	*CaNHX2*	Chr05	947	104045.39	5.44	0.242	Cell membrane.
Capana06g003039	*CaNHX3*	Chr06	527	58411.65	8.79	0.524	Vacuole
Capana08g000122	*CaNHX4*	Chr08	1181	129915.14	6.26	0.090	Cell membrane
Capana08g000123	*CaNHX5*	Chr08	624	67507.96	7.62	0.619	Vacuole
Capana08g001332	*CaNHX6*	Chr08	482	53412.57	6.45	0.590	Vacuole
Capana10g000166	*CaNHX7*	Chr10	512	57296.70	8.59	0.452	Vacuole
Capana00g000346	*CaNHX8*	Chr00	459	51409.16	6.36	0.000	Vacuole
Capana00g004012	*CaNHX9*	Chr00	360	39805.86	6.63	0.720	Vacuole

### Phylogeny Analysis of *CaNHX* Genes With Different Species

**FIGURE 1:**
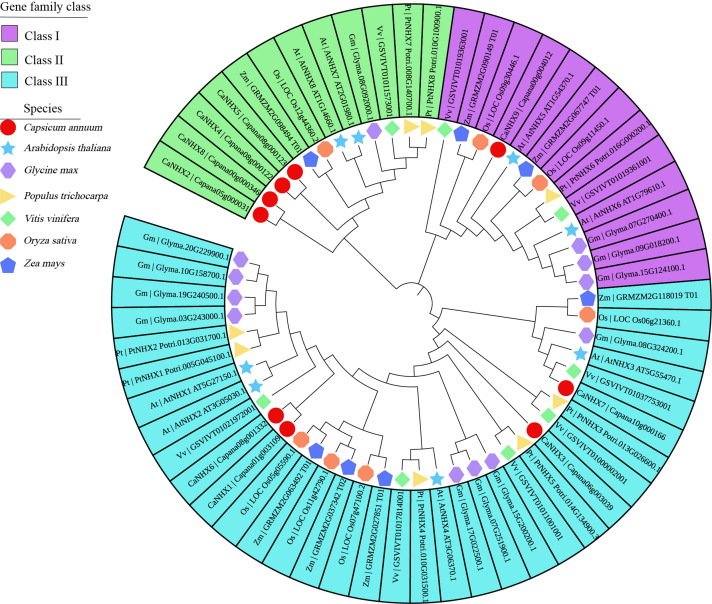
Phylogenetic analysis of *CaNHX* genes in *C. annuum.* Square: *C. annuum;* Star: *A. thaliana;* Horizontal hexagon: *G. max;* Right triangle*: P. trichocarpa;* Rhombus*: V. vinifera;* Octagon*: O. sativa;* Up pointing pentagram*: Z. mays.*

The 9 *CaNHX* genes in *C. annuum* identified were compared with 8 *AtNHXs* in *A. thaliana*, 12 *GmNHXs* in *Glycine max*, 8 *PtNHX*s in *P. trichocarpa*, 8 *VvNHXs* in *V. vinifera*, 7 *OsNHX*s in *O. sativa*, 7 *ZmNHXs* in *Zea mays* (Figure 1 and Supplementary Table 3). According to the phylogenetic tree, *NHX* genes were divided into three subgroups, among which subgroup I contained the most genes. Subgroup I and Subgroup III contain four genes, respectively, while subgroup II contains only one gene.

### Gene Structure and Conserved Sequence of *CaNHX* Genes

**FIGURE 2:**
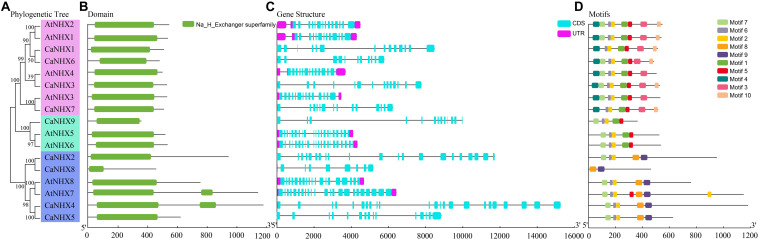
Gene structure and conserved sequence of *C. annuum* and *A. thaliana.* **(A)** Refined relationship between *C annuum* and *A. thaliana*. **(B)** Runctional structure domain. **(B)** NHX genes. **(C)** NHX genes structure. **(D)** Conservative motifs. **(D)** NHX genes. **(E)** Motifs.

**FIGURE 3:**
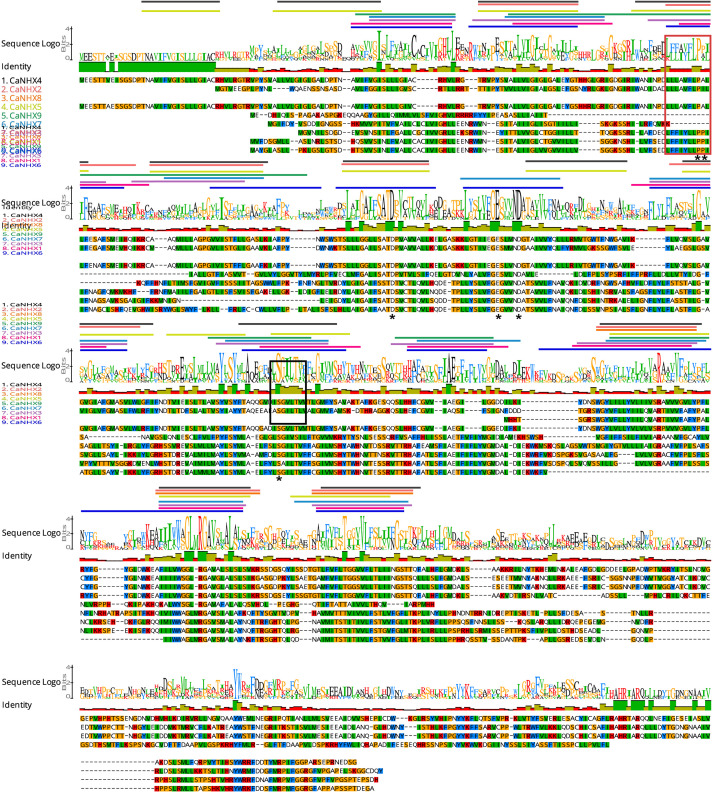
Sequence alignment of *CaNHX* Gene in pepper.

The *NHX* gene subfamily classification, gene structure, and motif analysis maps show the characteristics of *A. thaliana* and pepper gene families (Figure 2). Using TBtools to analyze the gene structure of 17 *NHX* genes family members in pepper and *A. thaliana*, the results showed that the exon number of the *CaNHX* gene family was mainly distributed between 8 and 24, while that of *AtNHX* gene family members in *A. thaliana* was between 12 and 23, among which the exon number of Class I subgroup was stable between 12 and 14, while the exon number of Class III was the largest. The NHX protein sequences of *C. annuum* and *A. thaliana* were analyzed by MEME web tool. According to the distribution of motif of *CaNHX* genes family members, the motif number is consistent with the phylogenetic tree. For example, in the Class I subfamily, there are 5 motif sequences, which are motif f1, motif2, motif5, motif6, and motif7. It is consistent with the phylogenetic tree classification of the Class I subgroup. The motif of the *CaNHX* gene in the Class II subfamily all contained motif8 and motif9, of which CaNHX8 was the one with the least motif number. In the Class III subfamily, the motif number of CaNHX1, CaNHX3, CaNHX6, and CaNHX7 remained at 7–8, of which motif8 and motif9 did not exist in the Class III subfamily. Motif7 is a typical amiloride-binding site (LFFIYLPPI), which is a motif contained in the genes of salt-tolerant plants and transgenic *NHX* plants, and contains the motif in CaNHX2, CaNHX3, CaNHX4, and CaNHX5 (Figures 2, 3).

### Chromosome Localization, Collinearity Analysis, and Ka/Ks Analysis

**FIGURE 4:**
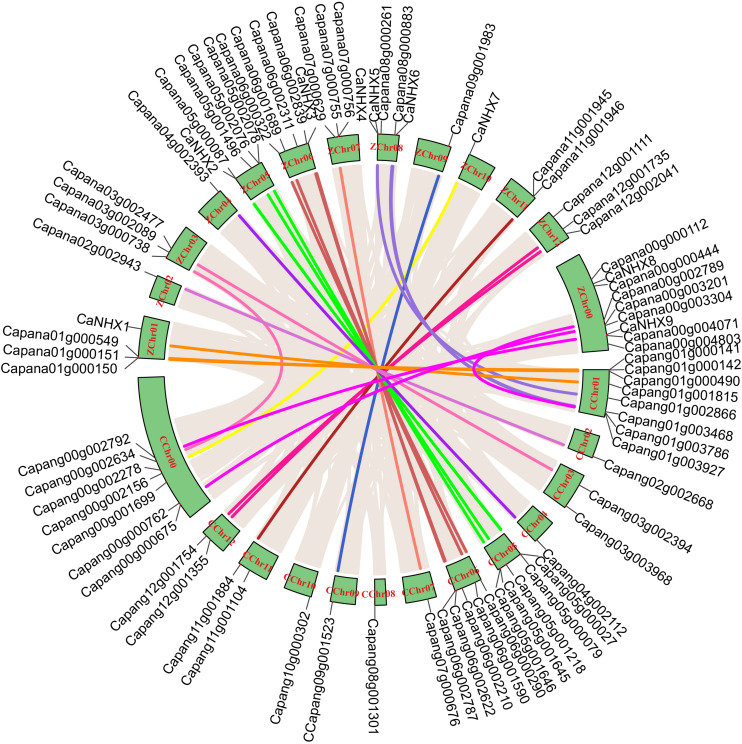
Chromosome location and collinearity analysis of *CaNHX* genes in pepper.

**FIGURE 5:**
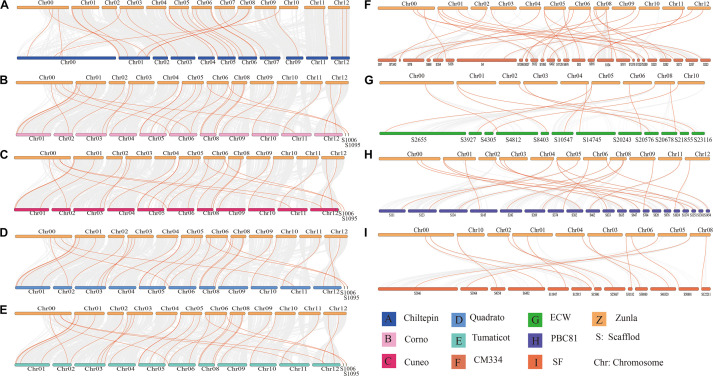
Collinearity analysis of *CaNHX* gene in pepper. **(A)** Chiltepin; **(B)** Corno; **(C)** Cuneo; **(D)** Quadrato; **(E)** Tumaticot; **(F)** CM334; **(G)** ECW; **(H)** PBC81; **(I)** SF. Z: Zunla-1.

Through identification, 9 were identified in cultivated pepper (Zunla-1) and 6 were identified in wild pepper (Chiltepin) (Supplementary Tables 5, 6). According to the gene sequence, 9 *CaNHX* gene sequences were mapped to 5 chromosomes, among which one was a simulated chromosome (not assembled to chromosome), 3 *CaNHX* genes were mapped to Chr08, but 2 genes were not mapped to the chromosome, and the other chromosomes Chr01, Chr05, Chr06, and Chr10 were all one *CaNHX* gene (Figure 4). To study the whole genome duplication (WGD) event, 42 cultivars were identified (Contains the confirmed 9 *CaNHX* genes) by Hmmsearch in cultivated pepper and 37 cultivars identified by hmmsearch in wild pepper were analyzed together (Supplementary Table 3). There are more collinearity relationships among the 9 pepper genes identified, among which *CaNHX4* and *CaNHX6* are on Chr08 of Zunla-1, while the collinearity block of this gene is on wild type Chr01. There is a collinearity block between *CaNHX*7 and wild-type Chr00 on Zunla-1 chromosome Chr10. Among them, *CaNHX*1, *CaNHX*5, and *CaNHX*8 have no collinearity block relationship, while *CaNHX*2, *CaNHX*3, and *CaNHX*9 also have collinearity, but their chromosomal positions do not change. The results indicated that the cultivated pepper Zunla-1 and the wild pepper were from the same ancestor, and there was a certain gene replication event. At the same time, we performed collinearity analysis on the genome of pepper varieties with Zunla-1and other pepper genomes, including Chiltepin, Corno, Cuneo, Quadrato, tumaticot, CM334, ECW, PBC81, and SF (Figure 5). The collinearity analysis between Zunla-1 and Corno, Cuneo, Quadrato, and Tumaticot showed that the chromosomal position relationship of Corno, Cuneo, Quadrato, and Tumaticot was the same. The results showed that the four pepper varieties were derived from the same ancestor and had less variation during the species evolution. However, the position relationship between the four pepper varieties and Zunla-1 changed greatly, which indicated that they had a far evolutionary relationship with more variation. Zunla-1 showed significant variation with Chiltepin, CM334, ECW, PBC81, and SF, and the changes of gene position were obvious, indicating that Zunla-1 was far related to the other five varieties of pepper. Three pairs of homologous loci were obtained by analyzing the Ka/Ks ratio of the *CaNHX* gene, and their Ka/Ks were less than one, indicating that the gene was mainly purified during the evolution of the *CaNHX* gene in pepper (Table 2).

**TABLE 2:** Nucleotide substitution rate of Pepper *CaNHX* gene.

Collateral homologous gene site	Non-synonymous substitution rate (Ka)	Synonymous substitution rate (Ks)	Selective pressure ratio (Ka/Ks)
*CaNHX1*-*CaNHX6*	0.201701088	2.493573377	0.080888371
*CaNHX2*-*CaNHX8*	0.026980027	0.041710828	0.646835085
*CaNHX4*-*CaNHX5*	0.155507334	0.327196446	0.475272076

### Promoter Analysis of *C. annuum CaNHX* Genes

**FIGURE 6:**
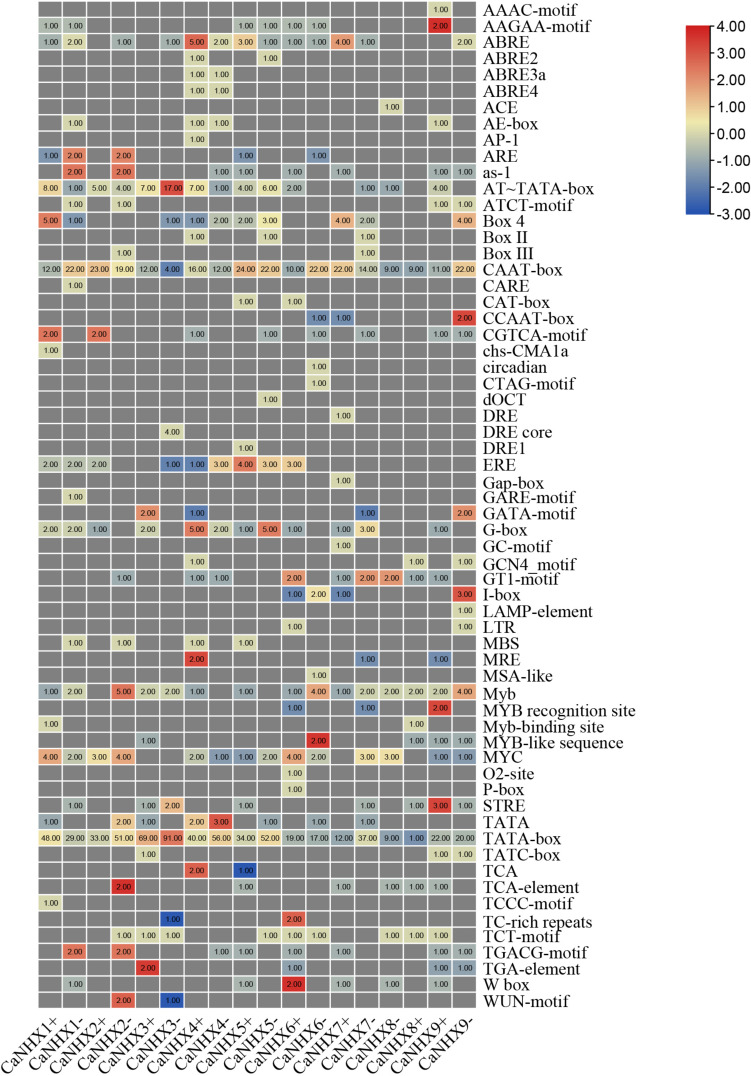
*Cis*-acting elements of *C. annuum CaNHX* genes.

By analyzing the upstream 2,000 bp sequence of the *CaNHX* genes, the *cis*-acting elements of the gene were predicted. In addition to a large number of basic elements—CAAT-box and TATA-box—there are also G-Box, GAG-motif, chs-CMA1a, TCT-motif, GATA-motif, GT1-motif, and AE-box in the *CaNHX* gene family, and TCA-elements in the salicylic acid reaction. Also present were *cis*-acting elements, TC-rich repeats, meristem expression elements, CAT-box, MYB binding site elements, MBS, MeJA response elements, GCTCA-motif, auxin response elements, TGA-elements, etc. (Figure 6 and Supplementary Table 7). The analysis showed that *CaNHXs* may be regulated by such things as light and salicylic acid, and may participate in defense mechanisms through these *cis*-acting elements, thus playing a role in protecting plant growth.

### Co-expression Network of *CaNHX* Gene Under Stress Treatment

**FIGURE 7:**
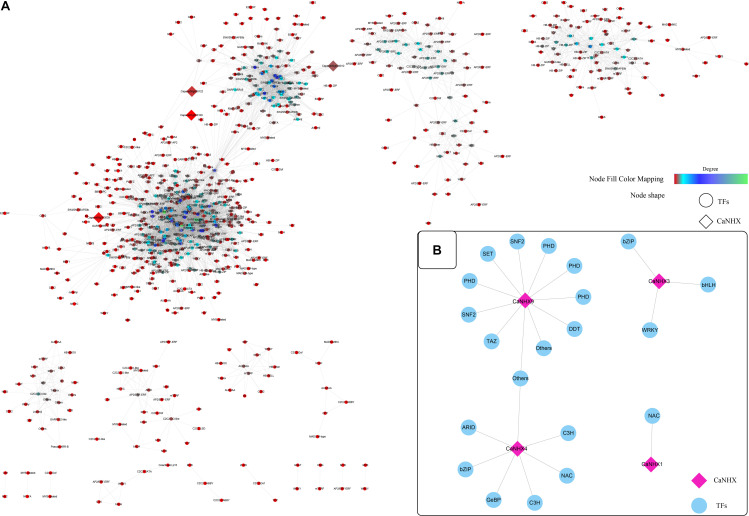
**(A,B)** Co-expression network of *C. annuum CaNHX* gene under NaCl stress (Diamond: CaNHX genes; Circular: TFs; Node Fill Color Mapping: Degree).

According to established methods, the co-expression network related to the *CaNHX* gene was extracted. Under stress, 10 groups of data were obtained: ABA, IAA, GA3, SA, JA, sodium chloride, mannitol, hydrogen peroxide, heat stress, cold stress, plus control groups. The co-expressed gene of the *CaNHX* gene was extracted by script, and the co-expressed gene related to the *NHX* gene under stress was obtained (Figure 7 and Supplementary Table 8). According to the co-expression network, in the control group, the genes co-expressed with the *NHX* gene contained fewer co-expression network genes than the other 10 groups, among which ABA, IAA, GA3, and mannitol were the most abundant. The number of co-expression genes was the highest under heat stress and cold stress, but other co-expression genes were also present under NaCl stress. Under salt stress, a total of 4 *CaNHX* genes were co-expressed with transcription factors, among which *CaNHX9* co-expressed the most with 11 transcription factors, followed by *CaNHX4* with 7 transcription factors, and *CaNHX1* with only 1 NAC co-expressed with *CaNHX1* was the least.

### Expression Pattern of Pepper *NHX* Genes Under Hormone and Abiotic Stress

**FIGURE 8:**
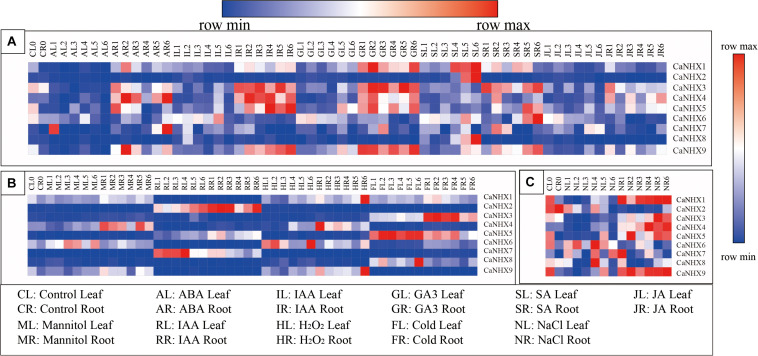
Expression pattern of *CaNHX* gene in pepper under different stress in root and leaf. **(A)** Hormonal stress. **(B)** Abiotic stress. **(C)** Salt stress.

The expression profile of the *CaNHX* gene in pepper was analyzed using an online database (Figure 8). The results showed that the expression of *CaNHX3*, *CaNHX4*, and *CaNHX5* in roots was upregulated compared with that in leaves during IR (IAA root stress) and GR (GA3 root stress), while the expression of *CaNHX2*, *CaNHX6*, *CaNHX7*, *CaNHX8*, and *CaNHX9* was lower in leaves. Under hormone stress, *CaNHX1* expression was upregulated. Under abiotic stress, *CaNHX1* and *CaNHX9* genes were upregulated in HR (heat root stress), while *CaNHX2*, *CaNHX3*, and *CaNHX4* were upregulated in RR (H_2_O root stress), FR (cold root stress), and MR (mannitol root stress), whereas *CaNHX5* was upregulated in FL (cold leaf stress), *CaNHX6* was upregulated in HL (heat leaf stress), *CaNHX7* was upregulated in RL (H_2_O leaf stress), *CaNHX7* was upregulated in RL and *CaNHX8* in FL. At the same time, expression analysis of the *CaNHX* gene family under salt stress showed that *CaNHX1*, *CaNHX3*, *CaNHX4*, *CaNHX*5, and *CaNHX*9 were upregulated in roots, and their expression tended to be consistent over time. *CaNHX6*, *CaNHX7*, and *CaNHX8* were upregulated in NL (NaCl leaf stress), while the root expression of *CaNHX2* was higher in the blank control than in the leaves.

### Quantitative RT-PCR Analysis

**FIGURE 9:**
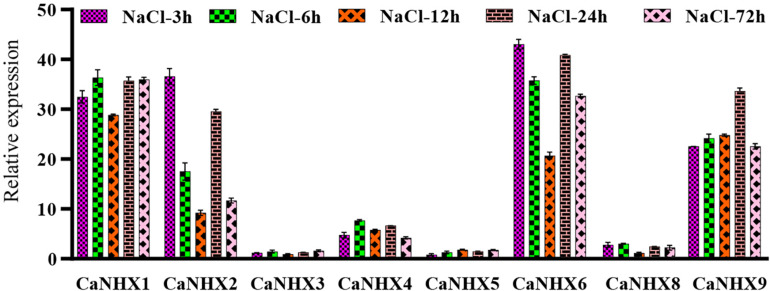
Quantitative RT-PCR analysis of pepper *CaNHX* gene.

The pepper at the 6-leaf growth stage was subjected to 100 Mmol salt stress, and the samples at different times (3, 6, 12, 24, and 72 h) were taken for fluorescence quantitative analysis (Figure 9). Results show that, under salt stress, when processing *CaNHX1*, *CaNHX2*, *CaNHX6*, *CaNHX9* showed a trend of increased expression, the five time node, *CaNHX1*, *CaNHX9* two genes in a state of relative balance, express no obvious floating. The expression of *CaNHX2* and *CaNHX6* began to be down-regulated over time after initial stress treatment and then began to be up-regulated after 24 h. The expression of *CaNHX3*, *CaNHX4*, *CaNHX5*, and *CaNHX8* genes was less obvious than others. *CaNHX1* with *CaNHX9* fluorescence quantitative results agree with the transcriptome data, in response to salt stress were a higher expressed state, *CaNHX2*, *CaNHX6*, *CaNHX7*, *CaNHX8* increase is not obvious in the transcriptome, which no expression in fluorescence quantitative *CaNHX7* gene, do not make the same amount in the transcriptome. In conclusion, two genes, *CaNHX1* and *CaNHX9*, were stably expressed in pepper under salt stress, which was consistent with transcriptome results. It was speculated that pepper could adjust its own ion balance by up-regulating the expression of *CaNHX1* and *CaNHX9* genes in the process of salt stress, so that pepper could adapt itself to the changes in the environment.

## Discussion

### *NHX* Gene Family in Pepper

We identified 9 *CaNHX* genes in the pepper genome (zunla-1), 6 in *C. annuum* chiltepin, 9 in *C. chinense*. PI159236, and 9 in *C. annnuum*. Cv.CM334. There were 9 identified in *C. nnuum*. Cv. ECW, 10 identified in *C. accatum*. PBC81, 8 identified in *C. nnuum*. SF, 8 identified in Corno, 8 identified in Cuneo, 9 identified in Quadrato, and 8 identified in Tumaticot. The *CaNHX* gene was found to contain up to 9 genes in different varieties of pepper. So far, *NHX* genes have been identified in many species, with the most identified in *Gossypium hirsutum* and *G. barbadense* (23 *GhNHX* and 24 *GbNHX*, respectively) (Fu et al., 2020).

However, in subcellular localization, *NHX* genes can be classified into three subgroups according to their subcellular localization according to previous reports which were divided into three categories, namely Vac-Class, Endo-class, and PM-class, among which VAC-class is located in Vacuole, Endo-class in Endoretinal Reticulum, and PM-class in Plasma membrane (Wu et al., 2019). Other *NHX* gene species based on subcellular localization also include *A. thaliana*, *P. trichocarpa*, *G. hirsutum*, *V. vinifera*, *Triticum aestivum*, *Oryza sativa*, *Sorghum bicolor*, *Cucurbita maxima*, *Solanum lycopersicum*, *Panicum virgatum*, *Eutrema halophilum*,*Spinacia oleracea*, and *Hordeum vulgare*. According to the classification of grapes by Ayadi et al. (2020) the *VvNHX* genes of grapes are divided into two categories, namely Group I Vacuolar (*VvNHX1–VvNHX5*) and Group II Endosomal (*VvNHX6*). However, *CaNHX* genes in pepper are not classified according to their subcellular location. *CaNHX2* and *CaNHX4* are located in the Cell membrane, while the other subcellular locations of *CaNHX* gene family members are located in Vacuole. There is no PM-Class and Endo-class in pepper, which is quite different from previous studies.

The *CaNHX* gene in Zunla-1 can be divided into three subfamilies, namely Class I, Class II, and Class III. In the identified *NHX* gene families, *NHX* contains a complete functional domain. These *CaNHX* genes can be divided into 3 categories, which are the same as *A. thaliana*, beet, and other plants reported by predecessors (Wu et al., 2019). In addition to *CaNHX*7, *CaNHX*8, and *CaNHX*9, the other six genes also have the typical amilorid-binding site of the *NHX* gene (FFI/LY/FLLPPI), but this structure does not exist in the Class II subgroup. At the same time, the intermediate residues LF/AV/IY, LF in Class I, LA in Class II, and IY in Class III, *PgNHX* gene also had the same motif and residues in pomegranate. Not only pepper but also *A. thaliana* had an *NHX* gene without an amiloride-binding site (Counillon et al., 1993; Dong et al., 2021). In the wheat *TaNHX* gene, it was found that under salt stress, the expression of *TaNHX2* and *TaNHX3* genes were higher in leaves and roots, and the expression of *TaNHX1* was higher only in roots. All three *TaNHX* genes contained an amiloride-binding site (LFFIYLLPPI) (Brini et al., 2005; Yu et al., 2007; Lu et al., 2014). It is speculated that the *NHX* gene containing an amiloride-binding site is more suitable for the growth of salinization conditions.

Plants are affected by the external environment during their growth, such as abiotic stress, drought, high temperature, salt, and alkali, etc. Transcription is particularly important in the response of plants to environmental changes. There are many *cis*-acting elements in the pepper *CaNHX* genes family, such as hormones and stress elements. Studies show that stress-related elements (such as high temperature, low temperature, drought, injury, and defense) and hormone-related elements (such as Auxin, Ethylene, GA, SA, MeJA, and ABA) are identified in the promoter of *PtNHXs*, and there are also *cis*-acting elements such as ABA and ABRE in sugar beet, which indicate that they can pass through during plant growth (Tian et al., 2017; Wu et al., 2019). In the transcriptional data of pepper, it was found that the co-expression networks of pepper under biotic and abiotic stress had higher gene network abundance than those under untreated conditions. In the co-expression network, *CaNHX*1, *CaNHX*3, *CaNHX*4, and *CaNHX*9 were found to be co-expressed with transcription factors, among which *CaNHX*3 was co-expressed with transcription factor WRKY, indicating that the *cis*-acting element of *CaNHX*3 was G-box, which has been found in studies. The G-box is an element associated with WRKY transcription factors under stress conditions. *CaNHX*3 was upregulated in several periods.

### Expression Profiles of *NHX* Genes in Pepper

Up to now, the function of the *CaNHX* gene in pepper has not been analyzed, and no report on the *CaNHX* gene in pepper has been reported. With the transformation of salt-tolerant transgenic plants, the *NHX* gene will provide more benefits for agricultural development in soil salinization. Transgenic technology has become one of the important ways to obtain salt-tolerant plants and verify gene function (Dhankher and Foyer, 2018). *NHX* can improve the salt tolerance of transgenic plants, and the overexpression of *AtNHX5* in rice can improve the salt tolerance and drought tolerance of transgenic rice, and the survival rate is higher (Li et al., 2011). *SbNHXLP* can improve the salt tolerance of tomatoe, and the Na^+^ level in tomato is lower, and the Ca^2+^ level is higher, compared with wild-type plants. *SbNHXLP* maintained ion homeostasis in tomato and alleviated NaCl stress (Kumari et al., 2017). When plants are subjected to salt stress, due to the lack of *NHX* expression to maintain homeostasis, the premature apoptosis of plants is caused, and the growth of plants is inhibited, thus affecting the yield of plants. Cao et al. (2011) through the overexpression of *TaNHX2*, improve the survival time of transgenic plants, showing salt tolerance. The number of flowering was more than that of the control group (Cao et al., 2011). In recent years, with the further exploration of the function of *NHX*, it has been found that *NHX* is resistant to cadmium, and the overexpression of *GmNHX1* enhances the antioxidant capacity of plants and reduces the absorption of cadmium (Yang et al., 2017). It was also found that silencing genes in plants had a great influence on plant growth and development. Rodríguez-Rosales et al. (2008) found that tomato seedling growth, fruit, and seed yield had significant inhibitory effects by silencing tomato *LeNHX2*. Overexpression of *LeNHX2* can enhance salt tolerance in plants (Rodríguez-Rosales et al., 2008; Baghour et al., 2019). In conclusion, overexpression of the *NHX* gene can improve ion homeostasis, osmotic regulation, reduce cell membrane damage, improve photosynthetic capacity, and play a role in plant protection and yield increase. The *CaNHX* gene in pepper has never been published and identified before. This study can provide theoretical support for research on the salt tolerance of pepper.

We found that the expression of *CaNHXs* was mainly concentrated in roots under hormone stress, while under abiotic stress, there were up-regulated expressions of *CaNHX2*, *CaNHX3*, and *CaNHX4* genes in roots. In the *cis*-acting elements of the *CaNHX* gene family, it was found that *CaNHX* was expressed under various hormones and stress. However, under salt stress, most of the *CaNHX* genes were up-regulated, which indicated that *CaNHX* genes in Pepper could condition its ion balance by expressing *NHX*. The function of plant vacuole *NHX* antiporter has been identified and expressed in an exogenous system to enhance the salt tolerance of plants. Akram et al. (2020) found that the *NHX* gene was up-regulated under salt stress, and Yokoi et al. (2002) found that *AtNHX1* had higher transcript abundance during salt stress (Yarra, 2019). The results indicated that the expression of *NHX* genes responded to salt stress during plant growth, which played a very important role in plant growth.

Transcriptome analysis found that the *CaNHX* gene had multiple expression patterns under single or multiple stress conditions. Meanwhile, the fluorescence quantitative verification in this study showed that the results were consistent with the transcriptome results, in which *CaNHX1* and *CaNHX9* were up-regulated under salt stress. These results indicated that the *CaNHX* gene provided a guarantee for the normal growth of pepper and the balance of ion channels of plant stress resistance.

## Conclusion

In the pepper genome, 42 *CaNHX* genes were identified by a Hidden Markov Model database search (hmmsearch). Of these, 9 genes with complete functional domains were identified by BLASTP. We constructed a phylogenetic tree and found that the 9 *CaNHX* genes were divided into three categories: Class I, Class II, and Class III. The exon number of the Class I subgroup was relatively stable, and the genes were distributed on six chromosomes; these were for hydrophilic proteins. There was a motif amilorid-binding site of the *NHX* gene (FFI/LY/FLLPPI) associated with salt tolerance in the pepper *CaNHX* gene. There are many elements in the *CaNHX* gene, such as hormone stress, salt stress, and so on, and it was found that the *CaNHX* gene is associated with many genes in the co-expression process, and salt stress conditions are also associated with many genes. Transcriptome analysis showed that the *CaNHX* gene was up-regulated under various abiotic stresses, which was verified in combination with fluorescence quantification in this study and found to be consistent with transcriptome results. In this study, the whole gene of Pepper was identified at the genome level, which provided a theoretical basis for the genetic breeding of pepper under stress.

## Data Availability Statement

The original contributions presented in the study are included in the article/Supplementary Material, further inquiries can be directed to the corresponding author/s.

## Author Contributions

XL, SY, ZZ, and CQ conceived and designed the experiments, and drafted the manuscript. YL, HQ, TL, JL, XC, XZ, YC, and JZ performed the experiments. SY and CQ analyzed the data. All authors read and approved the manuscript.

## Conflict of Interest

The authors declare that the research was conducted in the absence of any commercial or financial relationships that could be construed as a potential conflict of interest.

## Publisher’s Note

All claims expressed in this article are solely those of the authors and do not necessarily represent those of their affiliated organizations, or those of the publisher, the editors and the reviewers. Any product that may be evaluated in this article, or claim that may be made by its manufacturer, is not guaranteed or endorsed by the publisher.

## Footnotes

1
http://www.fao.org/

2
http://peppersequence.genomics.cn

3
http://pepper.snu.ac.kr/

4
https://www.pepper-genomics.unito.it/

5
http://pfam.xfam.org/

6
https://www.ebi.ac.uk/Tools/hmmer/

7
https://web.expasy.org/protparam/

8
http://www.csbio.sjtu.edu.cn/bioinf/plant-multi/

9
http://itol.embl.de/

10
https://www.ncbi.nlm.nih.gov/cdd/

11
http://gsds.gao-lab.org/

12
http://meme-suite.org/

13
https://github.com/arq5x/bedtools2

14
http://bioinformatics.psb.ugent.be/webtools/plantcare/html/

15
http://pepperhub.hzau.edu.cn/
